# Supplementary material for: An optimized multi-attribute decision-making approach to construction supply chain management by using complex picture fuzzy soft set
Source: PeerJ Comput Sci. 2023 Aug 30;9:e1540. doi: 10.7717/peerj-cs.1540 (PMC10495944; doi:10.7717/peerj-cs.1540)
Supplement: Supplemental Information 1 [file peerj-cs-09-1540-s001.docx]

Raw Data

**Manuscript Title: An optimized multi attribute decision making approach to construction supply chain management by using complex picture fuzzy soft set**

Following are the sources for the raw considered in the above-mentioned manuscript:

1. **Parameters (Source: Literature review)**
2. quality cum reliability
3. affordable cost
4. service cum processing
5. **Set of alternatives**
6. Supplier 1
7. Supplier 2
8. Supplier 3
9. **Softwares**
10. Microsoft Excel 2010
11. Microsoft Word 2010
12. WinEdt version Build: 20121130 (v. 7.0)
13. MathType version 6.9
14. **Company**

In this paper, a hypothetical name “BUILDCO” is used for company which wants to evaluate suppliers.

1. **References**

Total 49 references have been listed in the paper.

1. **Tables**

Only one Table is included in the paper.

1. **Figures**

Only one Figure is included in the paper.
