# Supplementary material for: An optimized multi-attribute decision-making approach to construction supply chain management by using complex picture fuzzy soft set
Source: PeerJ Comput Sci. 2023 Aug 30;9:e1540. doi: 10.7717/peerj-cs.1540 (PMC10495944; doi:10.7717/peerj-cs.1540)
Supplement: Supplemental Information 2 [file peerj-cs-09-1540-s002.docx]

**Code**

**Manuscript Title: An optimized multi attribute decision making approach to construction supply chain management by using complex picture fuzzy soft set**

In the above-mentioned manuscript, the authors have not used any machine learning tools or computer languages based softwares therefore there is no coding involved in the manuscript to design the algorithm. However, the methodological steps, without coding indices, in general template are being presented below that can be executed by any machine learning tool after transformation in codes:

1. Considering the essential sets and opinions of decision makers, construct cpFSS $\hat{\Pi}$, i.e.,

$$\hat{\Pi}=\left\{\left(\hat{a},\langle\alpha_{\hat{P}}(\hat{a})\exp^{j\beta_{\hat{P}}(\hat{a})},\alpha_{\widehat{Ne}}(\hat{a})\exp^{j\beta_{\widehat{Ne}}(\hat{a})},\alpha_{\hat{N}}(\hat{a})\exp^{j\beta_{\hat{N}}(\hat{a})}\rangle\right):\hat{a}\in\hat{\Lambda}\right\}.$$

1. Represent the cpFSS $\hat{\Pi}$ in matrix notation, where $p$and $q$ are the cardinalities of set of attributes and initial space of objects respectively.

$$\hat{℧}_{p\times q}=\left(\begin{matrix} \hat{\omega}_{11} & \hat{\omega}_{12} & \cdots& \hat{\omega}_{1q} \\ \hat{\omega}_{21} & \hat{\omega}_{22} & \cdots& \hat{\omega}_{2q} \\ \hat{\omega}_{31} & \hat{\omega}_{32} & \cdots& \hat{\omega}_{3q} \\ \vdots& \vdots& \ddots& \vdots\\ \hat{\omega}_{p1} & \hat{\omega}_{p2} & \cdots& \hat{\omega}_{pq} \end{matrix}\right)$$

Where

$$\hat{\omega}_{11}=\left\langle\alpha_{\hat{P}}(\hat{a}_{1})(\hat{s}_{1})\exp^{j\beta_{\hat{P}}(\hat{a}_{1})(\hat{s}_{1})},\alpha_{\widehat{Ne}}(\hat{a}_{1})(\hat{s}_{1})\exp^{j\beta_{\widehat{Ne}}(\hat{a}_{1})(\hat{s}_{1})},\alpha_{\hat{N}}(\hat{a}_{1})(\hat{s}_{1})\exp^{j\beta_{\hat{N}}(\hat{a}_{1})(\hat{s}_{1})}\right\rangle$$

$$\hat{\omega}_{12}=\left\langle\alpha_{\hat{P}}(\hat{a}_{1})(\hat{s}_{2})\exp^{j\beta_{\hat{P}}(\hat{a}_{1})(\hat{s}_{2})},\alpha_{\widehat{Ne}}(\hat{a}_{1})(\hat{s}_{2})\exp^{j\beta_{\widehat{Ne}}(\hat{a}_{1})(\hat{s}_{2})},\alpha_{\hat{N}}(\hat{a}_{1})(\hat{s}_{2})\exp^{j\beta_{\hat{N}}(\hat{a}_{1})(\hat{s}_{2})}\right\rangle$$

...............................................................................................

...............................................................................................

..............................................................................................

$$\hat{\omega}_{1q}=\left\langle\alpha_{\hat{P}}(\hat{a}_{1})(\hat{s}_{q})\exp^{j\beta_{\hat{P}}(\hat{a}_{1})(\hat{s}_{q})},\alpha_{\widehat{Ne}}(\hat{a}_{1})(\hat{s}_{q})\exp^{j\beta_{\widehat{Ne}}(\hat{a}_{1})(\hat{s}_{q})},\alpha_{\hat{N}}(\hat{a}_{1})(\hat{s}_{q})\exp^{j\beta_{\hat{N}}(\hat{a}_{1})(\hat{s}_{q})}\right\rangle$$

$$\hat{\omega}_{21}=\left\langle\alpha_{\hat{P}}(\hat{a}_{2})(\hat{s}_{1})\exp^{j\beta_{\hat{P}}(\hat{a}_{2})(\hat{s}_{1})},\alpha_{\widehat{Ne}}(\hat{a}_{2})(\hat{s}_{1})\exp^{j\beta_{\widehat{Ne}}(\hat{a}_{2})(\hat{s}_{1})},\alpha_{\hat{N}}(\hat{a}_{2})(\hat{s}_{1})\exp^{j\beta_{\hat{N}}(\hat{a}_{2})(\hat{s}_{1})}\right\rangle$$

$$\hat{\omega}_{22}=\left\langle\alpha_{\hat{P}}(\hat{a}_{2})(\hat{s}_{2})\exp^{j\beta_{\hat{P}}(\hat{a}_{2})(\hat{s}_{2})},\alpha_{\widehat{Ne}}(\hat{a}_{2})(\hat{s}_{2})\exp^{j\beta_{\widehat{Ne}}(\hat{a}_{2})(\hat{s}_{2})},\alpha_{\hat{N}}(\hat{a}_{2})(\hat{s}_{2})\exp^{j\beta_{\hat{N}}(\hat{a}_{2})(\hat{s}_{2})}\right\rangle$$

...............................................................................................

...............................................................................................

..............................................................................................

$$\hat{\omega}_{2q}=\left\langle\alpha_{\hat{P}}(\hat{a}_{2})(\hat{s}_{q})\exp^{j\beta_{\hat{P}}(\hat{a}_{2})(\hat{s}_{q})},\alpha_{\widehat{Ne}}(\hat{a}_{2})(\hat{s}_{q})\exp^{j\beta_{\widehat{Ne}}(\hat{a}_{2})(\hat{s}_{q})},\alpha_{\hat{N}}(\hat{a}_{2})(\hat{s}_{q})\exp^{j\beta_{\hat{N}}(\hat{a}_{2})(\hat{s}_{q})}\right\rangle$$

$$\hat{\omega}_{31}=\left\langle\alpha_{\hat{P}}(\hat{a}_{3})(\hat{s}_{1})\exp^{j\beta_{\hat{P}}(\hat{a}_{3})(\hat{s}_{1})},\alpha_{\widehat{Ne}}(\hat{a}_{3})(\hat{s}_{1})\exp^{j\beta_{\widehat{Ne}}(\hat{a}_{3})(\hat{s}_{1})},\alpha_{\hat{N}}(\hat{a}_{3})(\hat{s}_{1})\exp^{j\beta_{\hat{N}}(\hat{a}_{3})(\hat{s}_{1})}\right\rangle$$

$$\hat{\omega}_{32}=\left\langle\alpha_{\hat{P}}(\hat{a}_{3})(\hat{s}_{2})\exp^{j\beta_{\hat{P}}(\hat{a}_{3})(\hat{s}_{2})},\alpha_{\widehat{Ne}}(\hat{a}_{3})(\hat{s}_{2})\exp^{j\beta_{\widehat{Ne}}(\hat{a}_{3})(\hat{s}_{2})},\alpha_{\hat{N}}(\hat{a}_{3})(\hat{s}_{2})\exp^{j\beta_{\hat{N}}(\hat{a}_{3})(\hat{s}_{2})}\right\rangle$$

...............................................................................................

...............................................................................................

..............................................................................................

$$\hat{\omega}_{3q}=\left\langle\alpha_{\hat{P}}(\hat{a}_{3})(\hat{s}_{q})\exp^{j\beta_{\hat{P}}(\hat{a}_{3})(\hat{s}_{q})},\alpha_{\widehat{Ne}}(\hat{a}_{3})(\hat{s}_{q})\exp^{j\beta_{\widehat{Ne}}(\hat{a}_{3})(\hat{s}_{q})},\alpha_{\hat{N}}(\hat{a}_{3})(\hat{s}_{q})\exp^{j\beta_{\hat{N}}(\hat{a}_{3})(\hat{s}_{q})}\right\rangle$$

...............................................................................................

...............................................................................................

..............................................................................................

$$\hat{\omega}_{p1}=\left\langle\alpha_{\hat{P}}(\hat{a}_{p})(\hat{s}_{1})\exp^{j\beta_{\hat{P}}(\hat{a}_{p})(\hat{s}_{1})},\alpha_{\widehat{Ne}}(\hat{a}_{p})(\hat{s}_{1})\exp^{j\beta_{\widehat{Ne}}(\hat{a}_{p})(\hat{s}_{1})},\alpha_{\hat{N}}(\hat{a}_{p})(\hat{s}_{1})\exp^{j\beta_{\hat{N}}(\hat{a}_{p})(\hat{s}_{1})}\right\rangle$$

$$\hat{\omega}_{p2}=\left\langle\alpha_{\hat{P}}(\hat{a}_{p})(\hat{s}_{2})\exp^{j\beta_{\hat{P}}(\hat{a}_{p})(\hat{s}_{2})},\alpha_{\widehat{Ne}}(\hat{a}_{p})(\hat{s}_{2})\exp^{j\beta_{\widehat{Ne}}(\hat{a}_{p})(\hat{s}_{2})},\alpha_{\hat{N}}(\hat{a}_{p})(\hat{s}_{2})\exp^{j\beta_{\hat{N}}(\hat{a}_{p})(\hat{s}_{2})}\right\rangle$$

...............................................................................................

...............................................................................................

..............................................................................................

$$\hat{\omega}_{pq}=\left\langle\alpha_{\hat{P}}(\hat{a}_{p})(\hat{s}_{q})\exp^{j\beta_{\hat{P}}(\hat{a}_{p})(\hat{s}_{q})},\alpha_{\widehat{Ne}}(\hat{a}_{p})(\hat{s}_{q})\exp^{j\beta_{\widehat{Ne}}(\hat{a}_{p})(\hat{s}_{q})},\alpha_{\hat{N}}(\hat{a}_{p})(\hat{s}_{q})\exp^{j\beta_{\hat{N}}(\hat{a}_{p})(\hat{s}_{q})}\right\rangle\$.$$

1. Transform the matrix into core matrix

$$\hat{℧}_{p\times q}^{core}=\left(\begin{matrix} \hat{\omega}^{'}_{11} & \hat{\omega}^{'}_{12} & \cdots& \hat{\omega}^{'}_{1q} \\ \hat{\omega}^{'}_{21} & \hat{\omega}^{'}_{22} & \cdots& \hat{\omega}^{'}_{2q} \\ \hat{\omega}^{'}_{31} & \hat{\omega}^{'}_{32} & \cdots& \hat{\omega}^{'}_{3q} \\ \vdots& \vdots& \ddots& \vdots\\ \hat{\omega}^{'}_{p1} & \hat{\omega}^{'}_{p2} & \cdots& \hat{\omega}^{'}_{pq} \end{matrix}\right)$$

1. Split the core matrix into core matrix for amplitude terms and core matrix for phase terms as given below

$$\hat{℧}_{amp}^{core}=\left(\begin{matrix} \hat{\omega}^{''}_{11} & \hat{\omega}^{''}_{12} & \cdots& \hat{\omega}^{''}_{1q} \\ \hat{\omega}^{''}_{21} & \hat{\omega}^{''}_{22} & \cdots& \hat{\omega}^{''}_{2q} \\ \hat{\omega}^{''}_{31} & \hat{\omega}^{''}_{32} & \cdots& \hat{\omega}^{''}_{3q} \\ \vdots& \vdots& \ddots& \vdots\\ \hat{\omega}^{''}_{p1} & \hat{\omega}^{''}_{p2} & \cdots& \hat{\omega}^{''}_{pq} \end{matrix}\right)$$

where and

$$\hat{℧}_{pha}^{core}=\left(\begin{matrix} \hat{\omega}^{'''}_{11} & \hat{\omega}^{'''}_{12} & \cdots& \hat{\omega}^{'''}_{1q} \\ \hat{\omega}^{'''}_{21} & \hat{\omega}^{'''}_{22} & \cdots& \hat{\omega}^{'''}_{2q} \\ \hat{\omega}^{'''}_{31} & \hat{\omega}^{'''}_{32} & \cdots& \hat{\omega}^{'''}_{3q} \\ \vdots& \vdots& \ddots& \vdots\\ \hat{\omega}^{'''}_{p1} & \hat{\omega}^{'''}_{p2} & \cdots& \hat{\omega}^{'''}_{pq} \end{matrix}\right)$$

where

1. Compute maximum decision values, minimum decision values and score values for each alternative $\hat{s}_{m}, m \in\{1,2,...,q\}$ from matrix. Similarly compute the same values, and from matrix by using the following formulae:

1. Compute mean by using the following formula:

1. Select the alternative with maximum as optimal recommendation.
